# Arsenite Oxidation by a Newly Isolated Betaproteobacterium Possessing *arx* Genes and Diversity of the *arx* Gene Cluster in Bacterial Genomes

**DOI:** 10.3389/fmicb.2019.01210

**Published:** 2019-05-29

**Authors:** Melody Cabrera Ospino, Hisaya Kojima, Manabu Fukui

**Affiliations:** The Institute of Low Temperature Science, Hokkaido University, Sapporo, Japan

**Keywords:** arsenite oxidation, ARX, betaproteobacterium, *arx* gene cluster, freshwater, bacterial genomes

## Abstract

Microbes play essential roles in arsenic transformation in the environment. Microbial arsenite oxidation is catalyzed by either of two distantly related arsenite oxidases, referred to as AIO and ARX. The *arx* genes encoding ARX and its regulatory proteins were originally defined in the genomes of gammaproteobacteria isolated from an alkaline soda lake. The *arx* gene cluster has been identified in a limited number of bacteria, predominantly in gammaproteobacteria isolated from lakes characterized by high pH and high salinity. In the present study, a novel arsenite-oxidizing betaproteobacterium, strain M52, was isolated from a hot spring microbial mat. The strain oxidized arsenite under both microaerophilic and nitrate-reducing conditions at nearly neutral pH. Genome analysis revealed that the strain possesses the *arx* gene cluster in its genome and lacks genes encoding AIO. Inspection of the bacterial genomes available in the GenBank database revealed that the presence of this gene cluster is restricted to genomes of *Proteobacteria*, mainly in the classes *Gammaproteobacteria* and *Betaproteobacteria*. In these genomes, the structure of the gene cluster was generally well-conserved, but genes for regulatory proteins were lacking in genomes of strains belonging to a specific lineage. Phylogenetic analysis suggested that ARX encoded in the genomes can be divided into three groups, and strain M52 belongs to a group specific for organisms living in low-salt environments. The ArxA protein encoded in the genome of strain M52 was characterized by the presence of a long insertion, which was specifically observed in the same group of ARX. In clone library analyses with a newly designed primer pair, a diverse ArxA sequence with a long insertion was detected in samples of lake water and hot spring microbial mat, characterized by low salinity and a nearly neutral pH. Among the isolated bacterial strains whose arsenite oxidation has been demonstrated, strain M52 is the first betaproteobacterium that possesses the *arx* genes, the first strain encoding ARX of the group specific for low-salt environments, and the first organism possessing the gene encoding ArxA with a long insertion.

## Introduction

Despite their toxic nature, compounds of arsenic are utilized by some prokaryotes. Microbial transformation of arsenic includes respiratory As(V) reduction and As(III) oxidation for autotrophic growth, which are referred to as “arsenotrophy” (Oremland et al., 2009). The interconversion of arsenate and arsenite in arsenotrophy is catalyzed by one of the three enzymes of the dimethyl sulfoxide (DMSO) reductase family, arsenate reductase (ARR) and two distantly related arsenite oxidases, AIO and ARX (Zargar et al., 2012).

ARX is encoded by the *arxAB* genes, which were initially defined in *Alkalilimnicola ehrlichii* MLHE-1. This bacterium was isolated from anoxic bottom water of an alkaline-saline lake and can grow chemolithoautotrophically by anaerobic arsenite oxidation coupled with nitrate reduction (Oremland et al., 2002). The *arx* genes have also been identified in the genomes of phototrophic bacterial strains isolated from alkaline-saline environments, such as *Ectothiorhodospira* sp. PHS-1 and *Ectothiorhodospira* sp. BSL-9. These strains are capable of arsenite oxidation coupled to anoxygenic photosynthesis, which is referred to as photoarsenotrophy. The essential role of the *arxA* gene in chemoautotrophic and phototrophic arsenite oxidation was demonstrated by mutagenesis experiments with *A. ehrlichii* MLHE-1 and *Ectothiorhodospira* sp. BSL-9, respectively (Zargar et al., 2010; Hoeft McCann et al., 2017). In the genome of *A. ehrlichii* MLHE-1, the *arx* genes form a gene cluster consisting of structural and regulatory components. The structural component is comprised of five genes, *arxB2ABCD*. The other group of genes transcribed in the opposite direction, *arxXRS*, are thought to encode a regulatory system (Zargar et al., 2010). The same arrangement of the *arx* genes was also found in *Ectothiorhodospira* sp. PHS-1 (Zargar et al., 2012) and BSL-9 (Hernandez-Maldonado et al., 2016, 2017). To date, only a few arsenite-oxidizing bacteria have been reported to possess the *arx* genes or *arxA*. They are predominantly gammaproteobacteria isolated from lakes characterized by high pH and high salinity, including *Halomonas* sp. ANA-440 (Hamamura et al., 2014), *Halorhodospira halophila* SL1 (Challacombe et al., 2013; Hernandez-Maldonado et al., 2017), *Ectothiorhodospira* sp. MLW-1 (Hoeft McCann et al., 2017), and the three strains mentioned above. As an exceptional case, *Desulfotomaculum* sp. TC-1 (in the phylum *Firmicutes*) was isolated from a sulfidic hot spring at pH 5.5 (Wu et al., 2017).

As mentioned above, most current knowledge regarding ARX came from studies on gammaproteobacterial strains from environments with high pH and high salinity. On the other hand, however, the *arx* genes have also been identified in the genome of other bacteria whose ability for arsenite oxidation has not been demonstrated. One such strain, *Sulfuricella denitrificans* skB26, is a sulfur-oxidizing betaproteobacterium isolated from an artificial freshwater lake. In its genome, the *arx* genes form a gene cluster corresponding to that of *A. ehrlichii* MLHE-1. The protein encoded by the *arxA* gene of *S. denitrificans* skB26 is phylogenetically distinct from those of the halophilic and alkaliphilic gammaproteobacteria. In addition, it has a unique inserted amino acid sequence (Watanabe et al., 2014). In some previous studies, partial sequences of the *arxA* gene were obtained with culture-independent approaches. In one such study, *arxA* gene sequences closely related to that of *S. denitrificans* skB26 were frequently detected in samples characterized by low salinity and a nearly neutral pH (Ospino et al., 2018). These results suggest the presence of a lineage of ARX specific for freshwater environments, but arsenite oxidation by the ARX of *S. denitrificans* skB26 or other members of this lineage has not been demonstrated.

In this study, an arsenite-oxidizing betaproteobacterium possessing the *arx* genes was isolated. This isolate has the *arxA* gene belonging to the lineage frequently detected in freshwater environments. In addition, the diversity of the *arx* gene cluster was investigated by using genome sequences available in public databases.

## Materials and Methods

### Isolation of an Arsenite-Oxidizing Bacterium, Strain M52

To obtain a novel arsenite-oxidizing bacterium, a dark green microbial mat developed on an inclined concrete wall was obtained from the Jozankei hot spring located in Hokkaido, Japan. The mat is identical to that used in previous studies (Kojima et al., 2017; Ospino et al., 2018). A piece of mat was inoculated in a synthetic basal medium (pH 7.0 and 341.0 mg/L of NaCl), which was prepared as described previously (Connon et al., 2008). Prior to sterilization, the medium was purged with N_2_ or CO_2_ gas to enrich anaerobic organisms. Just before inoculation, stock solutions of sodium arsenite and sodium chlorate were added to the medium to final concentrations of 0.5 and 3 mM, respectively. The headspace of the bottle was filled with N_2_ gas, and incubation was performed in the dark at 32°C. The enrichment was subject to successive transfers to fresh media with an increased concentration of arsenite, 1 mM. During the enrichment culture procedure, the presence of organisms with the *arxA* gene was monitored by the PCR-based method previously described (Ospino et al., 2018). After the fifth transfer, a pure culture was obtained by repeated agar shake dilution (Widdel and Bak, 1992). For the isolation, another medium was used with Na_2_S_2_O_3_ and NaNO_3_ as an electron donor and acceptor, as described previously (Kojima et al., 2017).

### Genome Sequencing and Phylogenetic Analysis of Strain M52

Genomic DNA was extracted from strain M52 using a Wizard^®^ Genomic DNA purification kit (Promega, Madison, WI, United States). Genome sequencing and assembly were performed as described previously (Umezawa et al., 2016). The obtained genome was annotated with Rapid Annotation using Subsystem Technology (RAST) (Overbeek et al., 2014). The annotation overview of the genome was made in the SEED Viewer version 2.0 (online). The assembled genome was subject to inspection for genes involved in arsenic metabolism. The genome of strain M52 is available in GenBank under the Accession No. NZ_AP019373.

The closest cultured relatives of the novel strain were identified by comparison to the rRNA database in BLASTn, and a phylogenetic tree was constructed. The sequences were aligned with MAFFT version 7. Gaps and poorly aligned regions were excluded using Gblocks v0.91b. The tree topology was inferred by neighbor-joining using MEGA 6.

### Culturing Experiments With Arsenic

Arsenite oxidation ability of strain M52 and effects of arsenic on its growth were examined with culturing experiments at 45°C, as described below.

To verify whether strain M52 was capable of oxidizing arsenite anaerobically, culturing experiments were performed under anoxic conditions by using the basal medium used for the enrichment culture. The medium was supplemented with 0.2% (w/v) NaCl, 1–2 mg/L yeast extract, 0.5 mM sodium molybdate, 1 mM sodium arsenite and 10 mM sodium nitrate. For measurements of arsenite and arsenate concentrations, approximately 500 μL of medium was aseptically collected from each bottle at 0, 6, and 18 days of incubation. The collected samples were immediately filtered through 0.22 μm pore size filters and stored at -30°C until the measurements. The concentrations of arsenite and arsenate were determined by high-performance liquid chromatography (HPLC), as described previously (Watanabe et al., 2017).

Arsenite oxidation was also examined in the basically same medium which contained no nitrate, under anoxic, microoxic, and oxic conditions. For test of anoxic conditions, the medium was prepared as above, and headspace was filled with N_2_ gas. The medium for microoxic conditions were prepared as same as the above, but filter-sterilized air was added to the culture bottles after autoclaving, to obtain final oxygen concentrations of 1 or 2% (v/v) in the headspace. For oxic conditions, the medium was prepared without purging with N_2_ gas and head space was filled with air. Arsenite and arsenate were measured with HPLC just after inoculation and after 6 days incubation without shaking.

Effects of arsenic on growth were investigated under nitrate-reducing conditions, by monitoring turbidity along with concentrations of arsenate and nitrate. The experiments were performed with the medium same as that used for the first experiment, but concentration of yeast extract was 5 mg/L. Turbidity was measured as optical density at 660 nm, with a spectrophotometer. Arsenate and nitrate were quantified with ion chromatography equipped with conductivity detector (ICS-1500, Dionex), equipped with a column for anion analyses (IonPac AS12A, Dionex). The experiments were performed in the presence and absence of acetate, to assess heterotrophic and autotrophic growth. The experiment with acetate was started with medium containing 5 mM sodium acetate and 1 mM sodium arsenite. For comparison, bottles without arsenite were also prepared. In case of the experiments without acetate, the medium was initially supplemented with 0.65 mM sodium bicarbonate and 0.5 mM arsenite. These compounds were successively added to the medium during the incubation, by injection of dense solutions. At the latest stage of the experiments, 5 mM sodium acetate was added to the culture.

Tolerance to arsenite was tested in the basal medium, supplemented with sodium acetate (5 mM), sodium nitrate (5 mM), and 5 mg/L yeast extract. The strain M52 was cultured in presence of sodium arsenite with different concentrations (1, 2, 5, and 10 mM).

### Detection of the *arx* Gene Cluster in Prokaryotic Genomes

To explore the *arx* genes in prokaryotic genomes, amino acid sequences encoded by the *arxXRSB2ABCD* genes of *A. ehrlichii* MLHE-1 were used as queries to identify homologous proteins in the non-redundant protein database of the National Center for Biotechnology Information (NCBI)^1^. The identifications were made using Diamond v 0.9.14 (Buchfink et al., 2014), and two proteins were considered to be homologous based on the following thresholds: an amino acid identity of ≥ 30%, a spanning alignment of ≥ 60% of the length of the query, and an e-value ≤ 1e^-3^. In addition to sequences in the protein database, good-quality genome sequences without functional annotation were collected for the analysis from the “Microbial Genomes resource” in NCBI^2^. They were selected based on the following criteria: consist of contigs ≤ 300, have an N50 ≥ 20 kb and contain ≤ 10 kb of ambiguous base pairs. In the collected genomes, protein coding regions were deduced using Prodigal v2.6.3 in normal mode (with default parameters) (Hyatt et al., 2010), and the predicted proteins were used to build a custom database for identification of homologous proteins with the method described above. The data collection from NCBI databases occurred in January 2018. Furthermore, three genome sequences obtained in our laboratory were also included in the analysis. They are genomes of three strains isolated from the same microbial mat: strain M52 obtained in this study, *Sulfuritortus calidifontis* J1A (Kojima et al., 2017) and *Sterolibacteriaceae* bacterium strain J5B (Watanabe et al., 2019). They were analyzed with the same methods used for the unannotated genome sequences as described above.

### Phylogenetic Analyses

The identified proteins, homologous to ArxA of *A. ehrlichii* MLHE-1, were subjected to phylogenetic analysis, including the putative subunits of other enzymes from the DMSO reductase family. The amino acid sequences were aligned with MUSCLE (Edgar, 2004). Gaps were allowed within an appropriate block by Gblocks v0.91b (Talavera and Castresana, 2007). The tree was inferred with the distance criterion by the BioNJ algorithm using FastME 2.0 (Lefort et al., 2015). The inference included 1000 bootstraps replicates (Gascuel, 1997). Phylogenetic trees of proteins encoded by the *arxA, arxB*, and *arxD* genes were constructed as below. The amino acid sequences were separately aligned and curated as described above. The trees were reconstructed by the maximum-likelihood algorithm with the LG + G + I substitution model using PhyML (v3.0) (Guindon et al., 2010). The gamma shape parameter was estimated directly from the data. The aLRT test (SH-Like) was used to calculate the support values (Anisimova and Gascuel, 2006). To construct the ArxAB consensus tree, the alignments of ArxA and ArxB were concatenated using MEGA version 6 (Tamura et al., 2013) and then subjected to the remaining steps as explained above. All of the phylogenetic analyses were performed on the Phylogeny.fr platform (Dereeper et al., 2008).

Phylogenetic analysis of organisms possessing the *arx* genes was conducted on the basis of ribosomal proteins (RPs). The following 10 RPs were used for the analysis: L2, L3, L4, L13, L23, S2, S4, S9, S10, and S11 (see Supplementary Table S1 for detailed information). One of the identified genomes lacked some of these proteins, and thus it was excluded from the analysis. Each ribosomal protein was independently aligned using MAFFT (Katoh et al., 2017) with default parameters. The poorly aligned regions were removed using the Gblocks v0.91b program, allowing gaps located in less than 50% of the sequences. The resulting 10 alignments were concatenated using MEGA version 6. The tree was inferred by neighbor-joining under the p-distance model in MEGA 6. Support values were calculated using 1000 bootstrap replicates. The final trees were visualized using the R package ggtree (Yu et al., 2017).

### Identification of Conserved Motifs and Prediction of Secondary Structure

In the proteins encoded by *arxAB* genes, conserved motif regions were identified by Pfam online (Finn et al., 2014) and visualized in the multiple sequences alignment on MEGA 6. The presence and location of twin-arginine signal peptide cleavage sites were identified using the TatP 1.0 server online (Bendtsen et al., 2005). Secondary structure prediction was performed for the ArxA proteins with insertion, using the Jpred 4 server (Drozdetskiy et al., 2015).

### Clone Library Construction and Analysis of a Long Insertion in the *arxA* Gene

To explore the diversity of the ArxA proteins which have insertions, a set of degenerate primers was newly designed. The primers were designed based on the ArxA amino acid sequences characterized by the presence of a long insertion, encoded in the genomes of *S. denitrificans* skB26, *Sulfuritortus calidifontis* J1A, *Gammaproteobacteria* RIFOXYD12 FULL_61_37, *Gammaproteobacteria* RIFOXYA12 FULL_61_12, and *Betaproteobacteria* CG2_30_59_46. The primers were designed at positions flanking the insertion to obtain the full length of the insertion (Figure 8A). The primers are arxA_G2_F (AARCGTACCAAYCCSAAVAAGG) and arxA_G2_R (GTTCTTGGCGTAGTCRTCCAT). PCR was performed in a 25 μL volume reaction mixture containing 0.5 μmol/L of each primer, 1× Ex Taq Buffer (Takara, Shiga, Japan), 0.2 mmol/L dNTPs (Takara), 0.625 U of Ex Taq (Takara), 3% DMSO and template DNA solution. The PCR conditions were as follows: 94°C for 3 min; 34 cycles of denaturing at 94°C for 45 s, annealing at 55°C for 45 s, extension at 72°C for 1.5 min; and then a final extension step at 72°C for 7 min. Two DNA samples obtained in previous studies were used as templates for PCR amplification with newly designed primers: one water sample was obtained at a depth of 40 m in Lake Mizugaki (Watanabe et al., 2017), and a dark green microbial mat sample was retrieved from Jozankei hot spring (Ospino et al., 2018). The resulting PCR products were analyzed with agarose gel electrophoresis, and bands corresponding to expected sizes (ca. 1200 bp and ca. 900 bp, with and without insertion) were excised from the gel. From the pieces of gel, DNA was extracted with QIAquick Gel Extraction Spin Kit (Qiagen). The purified amplicons were cloned into pCR2.1-TOPO vector and transformed into TOP10 cells (Invitrogen), according to the manufacturer’s instructions. The resulting clone libraries were analyzed as described previously (Ospino et al., 2018), and operational taxonomic units (OTUs) were assigned at a cutoff value of 0.02 in Mothur (Schloss et al., 2009). The most abundant sequence of each OTU was selected as representative for phylogenetic analysis. The representative sequences were aligned with ArxA reference sequences using MUSCLE. The alignment was trimmed manually, excluding gaps. Phylogenetic relationships were inferred based on distance criteria using the BioNJ algorithm to construct a Neighbor-joining tree with 1000 bootstrap replicates in FastME 2.0. The ArrA of *Alkaliphilus oremlandii* OhILAs and *Halarsenatibacter silvermanii* were used as the outgroup. The nucleotide sequences obtained in this study have been deposited under the accession numbers LC439110 to LC439195.

### Phylogenetic Analysis of Partial Sequences of the *arxA* Gene Reported in Previous Studies

In some previous studies, partial sequences of the *arxA* gene were obtained with the primer pair arxA_Deg_F_B/arxA_Deg_R_B (Zargar et al., 2012; Hamamura et al., 2014; Wu et al., 2017) (Table 1). The phylogenetic positions of these sequences were reexamined with reference ArxA sequences collected in this study. In the phylogenetic analysis, partial sequences obtained in this study were also included. The alignment was made using MUSCLE and was trimmed manually to exclude the gaps. A neighbor-joining tree was reconstructed using the BioNJ algorithm with 1000 bootstrap replicates in FastME 2.0. The arsenate reductase ArrA sequences were used as the outgroup.

**Table 1:** Partial *arxA* gene sequences of uncultured organisms included in phylogenetic analysis in this study (Supplementary Figure S8).

Prefix	References	Characteristics of sampling site					Accession number
		pH	Temperature (°C)	Salinity (g/L)	Cl^-^ (mM)	Arsenic (μM)	
HC	Zargar et al., 2012	8.3^a^	Variable^a^	NA	0.6–0.8^a^	1.5–2.7	JN624760–JN624765
MLBX	Zargar et al., 2012	9.8b	NA	90^b^	500^b^	200	JN624766–JN624770
Paoha_Island _red_mat	Zargar et al., 2012	9.4	43	23	NA	100	JN624771
HJ	Hamamura et al., 2014	9.8	NA	6.2	NA	0.67–133	KC852945–KC852951
JZK1200	This study	7.8^c^	42.6^c^	NA	40.2^c^	40^c^	LC439110–LC439121
JZK900							LC439122–LC439154
MZG900	This study	7.0	10^d^	NA	1.4	1.5^d^	LC439155–LC439195

*Marked values were cited from references which are different from those for the sequences, as follows: ^*a*^Wilkie and Hering, 1998; ^*b*^Oremland et al., 1993; ^*c*^Ospino et al., 2018; ^*d*^Watanabe et al., 2017. NA, not available.*

## Results

### Isolation and Characterization of Arsenite-Oxidizing Bacterium Strain M52

From the microbial mat, arsenite-oxidizing enrichment cultures were established. The presence of an *arxA*-carrying organism in culture was confirmed with PCR amplification and sequencing with a previously reported primer pair. By analyzing the 16 rRNA gene, it was revealed that the cultures were dominated by a bacterium related to *Sterolibacteriaceae* bacterium strain J5B (Watanabe et al., 2019). The dominating organism was isolated by using the same methods that were used for the isolation of strain J5B, and the resulting isolate was designated strain M52. During the isolation procedures, strain M52 showed anaerobic and chemolithoautotrophic growth depending on nitrate and thiosulfate. The strain J5B is facultatively autotrophic bacterium which can grow on some organic acids (Watanabe et al., 2019). Among the organic substrates which support growth of strain J5B, acetate and lactate were tested with strain M52. Both these substrates supported anaerobic growth of strain M52 under nitrate reducing conditions.

**FIGURE 1:**
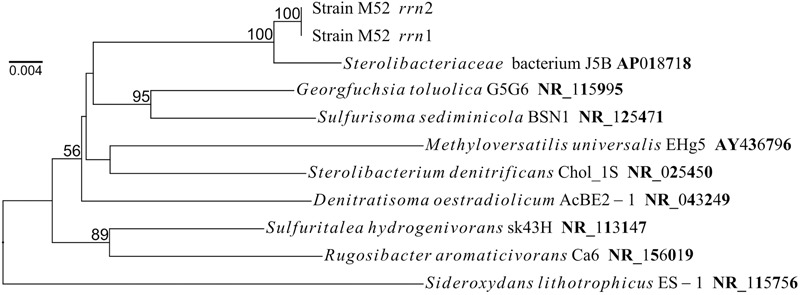
Phylogenetic position of two copies of the 16S rRNA gene in the genome of strain M52. Numbers on nodes represent percentage values (greater than 50%) of 1000 bootstrap resampling.

The complete genome of strain M52 consisted of a single circular chromosome with a length of 2.74 Mb and a G+C content of 63.6%. In the genome, 2794 coding sequences were predicted. There are two copies of the *rrn* operons which contain the 16S rRNA genes with slightly different lengths. BLASTn searches revealed that the most closely related species with validly published names were *Sterolibacterium denitrificans* and *Georgfuchsia toluolica*, with sequence identities of 94–95%. By constructing a phylogenetic tree, it was confirmed that strain M52 is a novel member of the family *Sterolibacteriaceae* (Figure 1).

In the genome of strain M52, the *arxA* gene was identified, and its nucleotide sequence perfectly matched the partial sequence detected in the enrichment cultures. The *arxA* gene of strain M52 encoded a protein that has 92 extra amino acids compared to that of strain MLHE-1, but the protein is similar to its homolog in *S. denitrificans* skB26. In addition to *arxA*, the other *arx* genes were also identified nearby *arxA*. Some differences in arrangement of these genes were observed, as described later in more detail.

Regarding other genes related to arsenic metabolism, strain M52 had genes for energy-dependent arsenic detoxification: *arsC* (detoxifying arsenate reductase), *arsR* (transcriptional repressor) and *acr3* (arsenite–specific efflux pump). On the other hand, the *arxM* gene, which is involved in arsenic methylation, was absent in the genome. The *aio* genes, which encode another type of arsenite oxidase, and *arrAB*, which encodes respiratory arsenate reductase, were not identified in the genome of strain M52.

Further inspection of the genome revealed that strain M52 has genes required for respiratory nitrate reduction. They include the genes encoding catalytic subunits of periplasmic nitrate reductase (*napA*), nitrite reductase (*nirS*), nitric-oxide reductase (*norB*) and nitrous oxide reductase (*nosZ*). The genome also harbors genes required for respiration with oxygen, the *ccoNOQP* genes encoding cytochrome *c* bb3-oxidase and the *cydAB* genes encoding cytochrome *bd* oxidase. Although the strain was isolated from enrichment culture with chlorate, genes for chlorate reduction were not identified in the genome.

### Arsenic Metabolism of Strain M52

**FIGURE 2:**
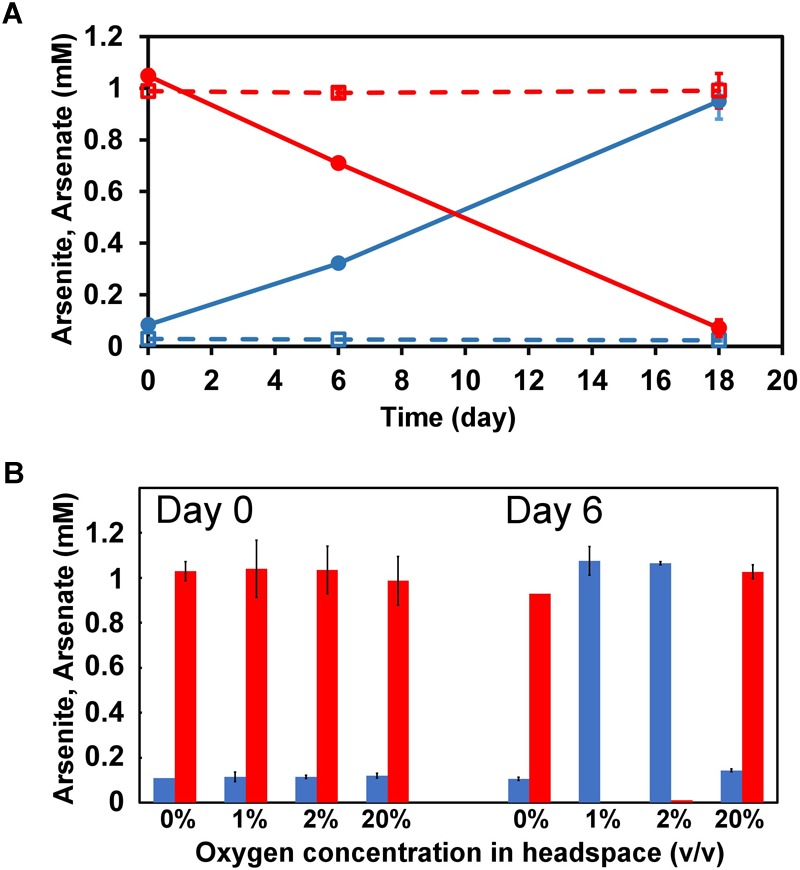
Arsenite oxidation to arsenate by strain M52. **(A)** Changes in concentrations of arsenite (red) and arsenate (blue) in medium containing nitrate, incubated under anoxic conditions. Error bars represent standard deviation from two independent assays. Dashed lines represent uninoculated control. **(B)** Effect of oxygen concentration in headspace on arsenite oxidation in medium without nitrate. Red and blues bars represent concentrations of arsenite and arsenate, respectively. Error bars represent standard deviation from triplicates (20%) or duplicates (the others).

Under anoxic conditions, strain M52 oxidized arsenite to arsenate in the medium containing nitrate as sole electron acceptor (Figure 2A). In the uninoculated control, changes in concentrations of arsenate or arsenite were not observed. Arsenite oxidation by strain M52 was also tested in the medium without nitrate. In the absence of nitrate, arsenite oxidation was not observed under anoxic conditions (Figure 2B). In the same medium, strain M52 oxidized arsenite when incubation was performed under microoxic conditions with 1 or 2% oxygen in the headspace (Figure 2B). The oxygen-dependent arsenite oxidation was not observed under atmospheric oxygen level (20%).

**FIGURE 3:**
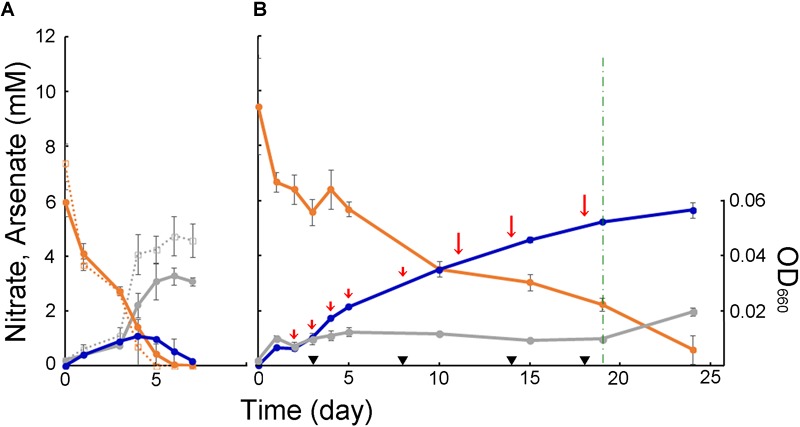
Anaerobic growth of strain M52 assessed as optical density at 660 nm (gray), and changes in concentrations of nitrate (orange) and arsenate (blue). Bars represent standard deviation from triplicates. **(A)** Experiments in medium containing acetate. Dashed lines represent culture without arsenite. **(B)** Experiments in medium without acetate. Short and long red arrows indicate time points of addition of arsenite, 0.5 and 1 mM, respectively. Black triangles and dashed green line indicate time points of addition of bicarbonate (0.65 mM) and acetate (5 mM), respectively.

To assess effects of arsenic on anaerobic growth of strain M52, cell density was monitored along with changes in concentrations of nitrate and arsenate. As shown in Figure 3A, 1 mM of arsenite showed negative effect on heterotrophic growth on acetate. In the presence of acetate, arsenate was generated in the early stage of growth, but it seemed to be reduced back into arsenite in the later stage of incubation possibly by the *ars* system. The effects on autotrophic growth was tested with gradual supplement of arsenite to abate toxicity (Figure 3B). With a serial addition of arsenite, continuous production of arsenate was observed throughout the incubation. A small increase in turbidity was observed until day 5, but no obvious growth was observed during the period from day 5 to day 19. In this period, supplement of inorganic carbon source did not enhanced growth. After the measurements at day 19, acetate was added to the culture. After that, cell density increased within the following 5 days, suggesting that viability was retained during the experiment (Figure 3B). In these experiments, nitrate was monitored with ion chromatography which can detect nitrite as well. In all samples analyzed, however, nitrite was not detected.

Arsenite tolerance of strain M52 was tested in presence of nitrate and acetate under anoxic conditions. Anaerobic growth on acetate was observed in the presence of 5 mM or lower concentration of arsenite, and higher concentrations resulted in slower growth of the strain. No growth was observed in the presence of 10 mM arsenite.

### Distribution, Composition, and Arrangement of the *arx* Gene Cluster

**FIGURE 4:**
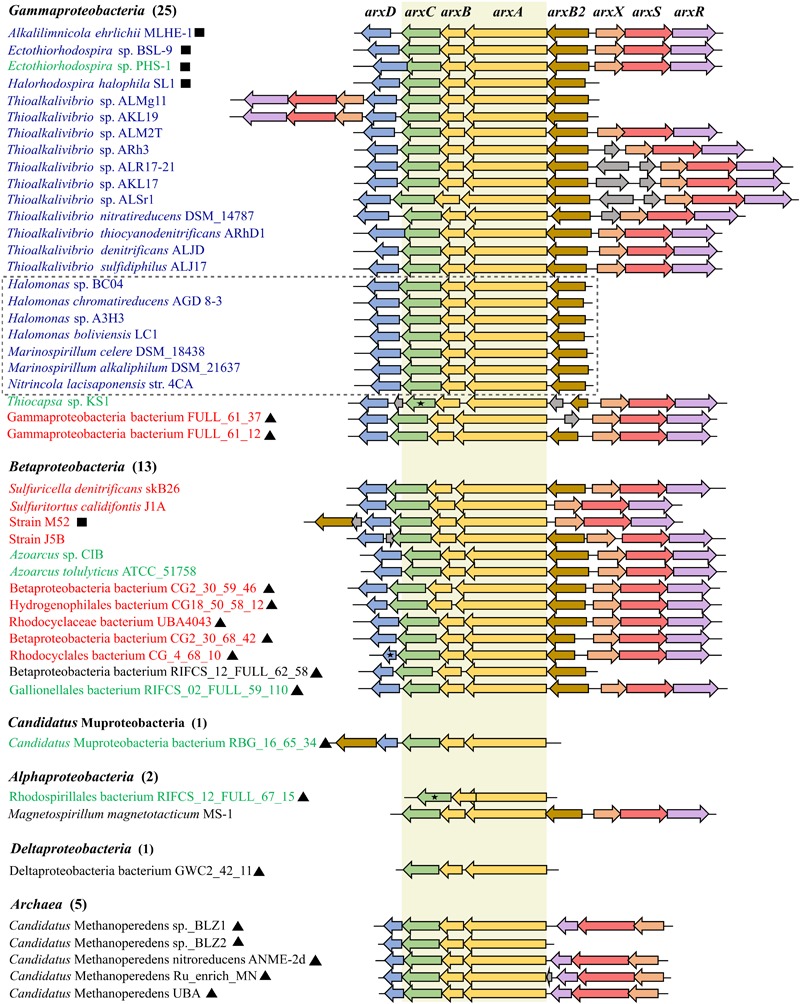
Arrangement of the *arx* gene cluster found in the genomes. Arrows with different colors represent the following genes: yellow for *arxAB*, green for *arxC*, blue for *arxD*, gold for *arxB2*, peach for *arxX*, coral for *arxS*, purple for *arxR*, and gray for hypothetical proteins. The number of genomes for each class-level taxonomy of bacteria is shown in parentheses. Name of organisms with ARX of group 1, group 2, and group 3 are shown in blue, green and red respectively. Names with black square correspond to organism reported as *arx*-arsenite-oxidizing bacterium, including strain M52. Names with black triangle represent the metagenome-assembled genomes (MAGs). Black star represents the genes encoding products annotated as partial protein. Names of organisms affiliated to the Order *Oceanospirillum* are enclosed in a gray dashed square. The conserved *arxABC* genes are highlighted in beige color.

By using the sequences of *A. ehrlichii* MLHE-1 as queries, a total of 46 genomes were identified to contain full-length genes homologous to the *arxAB* genes (see Supplementary Table S2 for more detail). Among them, five genomes were those of archaea, and the others were bacterial genomes. Detailed descriptions of the genomes are presented in Supplementary Table S3. All 42 bacterial genomes (41 identified and *A. ehrlichii*) were from organisms belonging to the phylum *Proteobacteria* (Figure 4). Among them, 25 are members of the class *Gammaproteobacteria*, including 15 strains in the family *Ectothiorhodospiraceae* (in the order *Chromatiale*). This group included four strains whose ability to oxidize arsenite was demonstrated: *A. ehrlichii* MLHE-1, *Halorhodospira halophila* SL1, *Ectothiorhodospira* sp. PSH-1, and *Ectothiorhodospira* sp. BSL9. The other 11 strains of this family were members of the genus *Thioalkalivibrio*. As another major group in the class *Gammaproteobacteria*, 7 strains were identified in the order *Oceanospirillales*. This group included the genera *Halomonas, Marinospirillum*, and *Nitrincola* (Figure 4). The majority of these gammaproteobacterial strains were isolated from high saline and/or high-pH environments, but two gammaproteobacterial genomes identified were reconstructed from metagenomic data of a groundwater sample (Supplementary Table S3). In the class *Betaproteobacteria*, six cultured strains possessed the genes, along with seven metagenome-assembled genomes (MAGs). The remaining bacterial genomes were affiliated with the classes *Alphaproteobacteria, Deltaproteobacteria*, and *Candidatus* Muproteobacteria (Figure 4). On the other hand, all archaeal genomes identified were those of *Candidatus* Methanoperedens in the order *Methanosarcinales.* They were MAGs from anaerobic bioreactors performing methane oxidation coupled to nitrate reduction (Supplementary Table S3).

**FIGURE 5:**
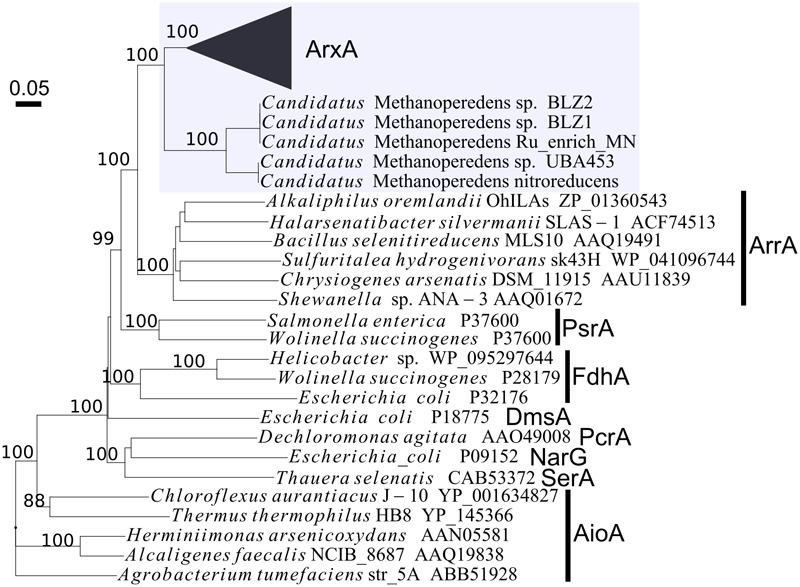
Phylogenetic positions of proteins homologous to ArxA of *Alkalilimnicola ehrlichi* MLHE-1, within the DMSO reductase family proteins. The ArxA of *A. ehrlichi* MLHE-1 and its homologs identified by BLAST searches are highlighted with a shaded box. An uncollapsed version of this tree is presented in Supplementary Figure S1. PsrA, polysulfide reductase; FdhA, formate dehydrogenase; DmsA, dimethyl sulfoxide reductase; SerA, selenate reductase; NarG, respiratory nitrate reductase; PcrA, perchlorate reductase.

In all the genomes identified, the *arxABC*-like genes were conserved in the same order and direction. As well, arrangement of genes around them showed significant similarities among the genomes (Figure 4). Among the genomes, 17 genomes had gene organization identical to that of the *arx* genes in *A. ehrlichii* MLHE-1 (Figure 4). For the proteins encoded by the gene homologous to *arxA*, a phylogenetic tree was constructed to identify their phylogenetic positions within the DMSO reductase family (Figure 5 and Supplementary Figure S1). In the tree, the proteins encoded in the identified genomes formed two distinct clades, corresponding to those of bacteria and archaea. The former encompasses ArxA of *A. ehrlichii* MLHE-1 and *Ectothiorhodospira* sp. PHS-1, which were included in the original definition of ARX. On the other hand, there is no evidence regarding the function of the proteins forming the latter cluster, and arsenic metabolism by *Candidatus* Methanoperedens has not been reported. Based on these results, the archaeal genomes were excluded from the following analyses. Hereafter, the bacterial genes homologous to the *arx* genes of *A. ehrlichii* MLHE-1 are simply referred to as *arx* genes.

The novel strain isolated in this study, strain M52, has *arx* gene cluster similar to that of *A. ehrlichii* MLHE-1, but the *arxB2* gene is located downstream of *arxABCD*, similar to a MAG of *Candidatus* Muproteobacteria (Figure 4). The *arxB2* gene was not identified in four bacterial genomes. The *arxD* gene was conserved in all the genomes except for three genomes from *Alphaproteobacteria* and *Deltaproteobacteria*.

Genes encoding regulatory proteins ArxXSR were commonly found upstream of the *arxB2ABCD* genes on the opposite strand. In the genomes of *Thioalkalivibrio* sp. ALMg11 and *Thioalkalivibrio* sp. AKL19, the *arxXSR* genes were identified downstream of *arxB2ABCD* on the same strand as exceptional cases (Figure 4). *Halorhodospira halophila* SL1 can oxidize arsenite, but the *arxXSR* genes were not identified in its genome. The *arxXSR* genes were consistently absent in the genomes of three genera in the order *Oceanospirillales* (*Halomonas* and *Marinospirillum* and *Nintricola lacisaponensis*) (Figure 4). The other genomes lacking *arxXSR* genes were distributed in *Candidatus* Muproteobacteria, *Alphaproteobacteria*, and *Deltaproteobacteria*.

### Phylogeny of Proteins Encoded by the Arx Proteins

**FIGURE 6:**
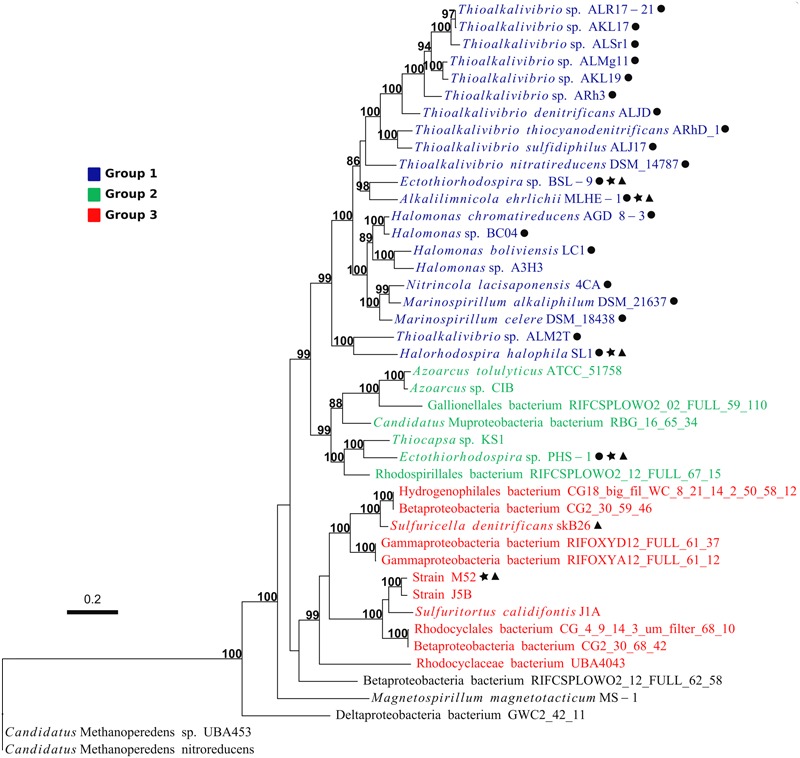
Phylogeny and grouping of ARX based on ArxAB sequences. Support values above 70 are shown. Names with: black circle, represent organisms isolated from high pH/high salinity environments; black star, represent arsenite-oxidizing bacteria; black triangle, correspond to organisms reported to hold an *arx* operon in the genome. Name of organisms with ARX of group 1, group 2, and group 3 are shown in blue, green and red respectively.

Phylogenetic relationships among the identified Arx proteins were further investigated in detail. At first, phylogenetic trees of ArxA and ArxB were separately constructed. The resulting trees showed phylogenetic congruence with minor exceptions, indicating the coevolution of these two subunits (Supplementary Figure S2). For a more reliable phylogenetic inference, an ArxAB consensus tree was constructed by concatenating the alignments of ArxA and ArxB (Figure 6). In the tree, the majority of ArxAB proteins were grouped into three groups corresponding to well-supported monophyletic clusters. In this study, these groups are referred to as group 1, group 2, and group 3, respectively (Figure 6). Correspondence between the grouping of ARX and the class-level taxonomy of the bacteria can be seen in Figure 4, 7. Direct comparisons between the phylogeny of bacteria (based on RPs) and ARX are shown in Supplementary Figure S3.

**FIGURE 7:**
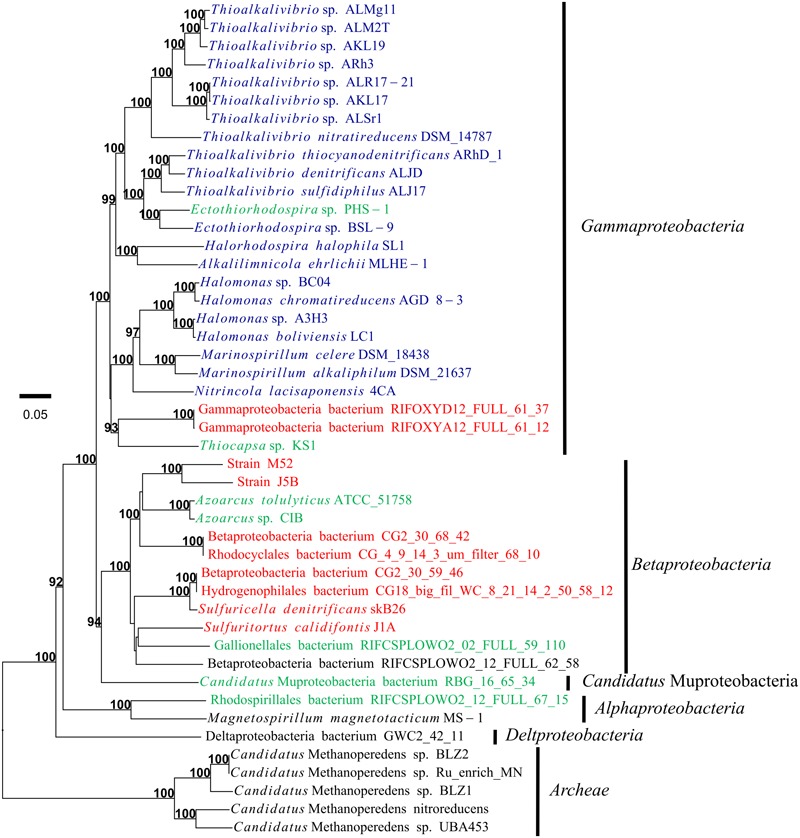
Phylogeny of the organisms possessing the *arx* genes, based on the concatenated amino acid sequences of 10 ribosomal proteins. Support values greater than 70% are indicated in bold. *Rhodocyclaceae* bacterium UBA4043 is not included due to absence of the majority of genes encoding ribosomal proteins. Name of organisms with ARX of group 1, group 2, and group 3 are shown in blue, green and red respectively.

The ARX of *A. ehrlichii* MLHE-1 was classified into the group 1. The ARX of this group was identified only in genomes of gammaproteobacterial haloalkaliphiles (Figure 3, 5). *Ectothiorhodospira* sp. PHS-1 is closely related to organisms with the ARX of group 1, but its ARX is phylogenetically distinct from those of the group 1. It was classified into group 2, together with the ARX of bacteria belonging to the classes *Gammaproteobacteria, Betaproteobacteria, Alphaproteobacteria*, and *Ca.* Muproteobacteria. The ARX of strain M52 belonged to group 3. This group consists of ARX encoded in bacteria isolated at a relatively low pH and low salt concentrations and MAGs from such environments.

A phylogenetic analysis of ArxD was also constructed (Supplementary Figure S4). In the tree, ArxD proteins associated with the ARX of group 1 and group 3 formed exclusive clusters. Phylogeny discordance with ArxD proteins was apparent in the ARX of group 2, but it was also observed in that of group 1. For instance, the positions of *Thioalkalivibrio nitratireducens* DSM 14787, *Thioalkalivibrio denitrificans* ALJD, and *Thioalkalivibrio* sp. ALM2T are considerably different in the phylogenetic trees of ArxD and ArxAB. In contrast, the phylogeny of ArxD proteins associated with ARX of group 3 was consistent with that of ArxAB, as indicated by the same branching pattern in the two trees (Supplementary Figure S4).

### Conserved Regions in the ArxAB Amino Acid Sequences

Comparison among amino acid sequences of the ArxAB encoded in the identified genomes indicated that they share some characteristics with those of *A. ehrlichii* MLHE-1 and *Ectothiorhodospira* sp. PHS-1 reported in previous studies (Zargar et al., 2010, 2012; Van Lis et al., 2013; Badilla et al., 2018), as described below. The ArxA sequences had three conserved regions commonly identified in subunit A of the DMSO reductase family enzymes: an iron–sulfur [4Fe-4S] motif; a catalytic binding pocket sequence similar to that suggested in the ArxA of *A. ehrlichii* MLHE-1 and *Ectothiorhodospira* sp. PHS-1 (Zargar et al., 2012); and a twin-arginine signal peptide on the N-terminus (Supplementary Figure S5). Additionally, all the sequences had an XGRGWG motif located near the putative catalytic binding pocket. This motif is argued to be one of the conserved motifs that distinguish ArxA from the closely related arsenate reductase ArrA, characterized by a corresponding motif of (R/K)GRY (Glasser et al., 2018). The ArxB also share similarities to subunit B of the enzymes from the DMSO reductase family with at least three conserved iron–sulfur [4Fe-4S] clusters (Supplementary Figure S6).

### Diversity of *arxA* With Insertion

**FIGURE 8:**
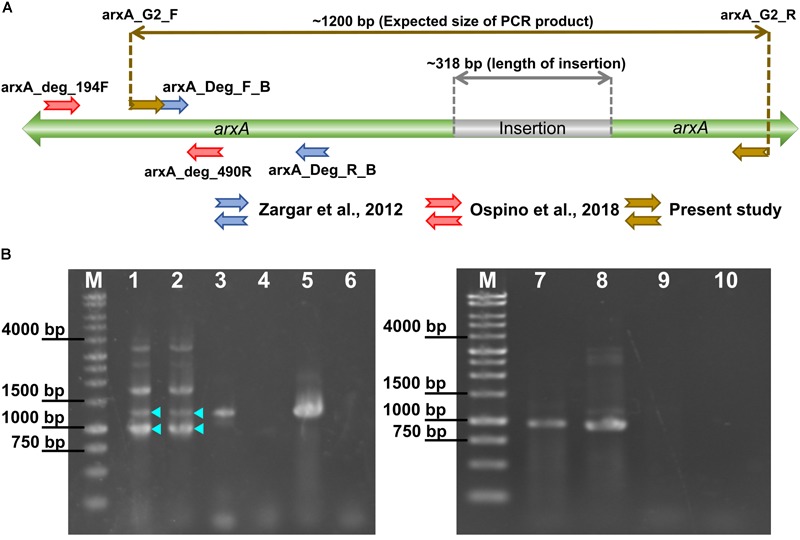
**(A)** Positions of PCR primers for the *arxA* gene and expected size of the PCR product. **(B)** Gel images showing the results of PCR with newly designed primers. Samples in lanes are: M, 10 kb DNA ladder as a size marker; (1–2) Jozankei microbial mat; (3) *Sulfuritortus calidifontis* J1A; (4) *Nitrincola lacisaponensis* 4CA; (5) *Sulfuricella denitrificans* skB26; (6) no template control; (7) Mizugaki water; (8) *Alkalilimnicola ehrlichii* MLHE-1; (9) *Sulfuritalea hydrogenivorans* sk43H; (10) no template control. In lanes 1 and 2, bands of expected sizes are marked with triangles.

As reported previously, ArxA of *S. denitrificans* skB26 has a long insertion (Watanabe et al., 2014). Similar insertions were identified in five other genomes, including strain M52 isolated in this study. To evaluate the diversity of the insertion in ArxA, a new primer pair was designed (Figure 8A). The primers were tested with purified genomic DNA from *S. denitrificans* skB26, *Sulfuritortus calidifontis* J1A, *A. ehrlichii* MLHE-1, and *Nitrincola lacisaponensis* 4CA. Among them, strains skB26 and J1A have the *arxA* gene with a long insertion. As a bacterium possessing the *arrA* gene, *Sulfuritalea hydrogenivorans* sk43H was used to confirm the specificity of the primer pair. The primer pair, named arxA_G2_F/arxA_G2_R, generated PCR products of approximately 1200 bp in size from strains skB26 and J1A (Figure 8B, lanes 3 and 5). A product of approximately 900 bp was obtained from strain MLHE-1 (Figure 8B, lane 8) but not from strain 4CA (Figure 8B, lane 4). No PCR products were obtained from the genomic DNA of strain sk43H (Figure 8B, lane 9). Although strains M52 and J5B have the insertions, their *arxA* gene sequences were not used for the primer design, because genomes of these strains were not available when the primers were designed. At a later time, the primer pair was tested with these strains but generated no PCR products.

Two environmental samples were analyzed with the new primer pair. They were water collected at a depth of 40 m in Lake Mizugaki (MZG) and a microbial mat obtained in Jozankei hot springs (JZK). A PCR product of the expected size with the insertion (1200 bp) was obtained only from the sample from JZK (Figure 8B, lanes 1 and 2). PCR amplicons of approximately 900 bp length were obtained in both JZK and MZG samples (Figure 8B, JZK: lanes 1 and 2; MZG: lane 7). With these PCR products, three clone libraries, named JZK1200, JZK900 and MZG900, were constructed (Table 1 and Supplementary Table S4). The phylogenetic tree in Supplementary Figure S7 shows the relationships between the partial ArxA sequences from the three libraries and the reference sequences. The neighbor-joining tree was reconstructed excluding gaps, using a final dataset of 310 compared amino acid positions. The tree indicated that five OTUs without insertions are clustered with ARX of group 2. The other OTUs were grouped with ARX of group 3. In the clone library of JZK1200, five OTUs with a long insertion were detected. Four of them are closely related to the ArxA of strains isolated from the same microbial mat (*Sulfuritortus calidifontis* J1A, strain M52 and strain J5B). The other OTU detected in this library was phylogenetically distinct from them and harbored the insertion with a clearly different sequence. In some OTUs detected in the library of 900 bp, insertions of another type were identified (Supplementary Figure S7). The insertions of this type, consisting of 12 amino acid residues, were also identified in ArxA of group 3 encoded in some MAGs.

The analysis of full ArxA sequences indicated that the insertions are distantly located from the putative functional conserved regions. No sequence motif was identified within the long insertion, but some amino acids were abundant and a few of them were conserved, such as lysine, alanine, glutamate, and leucine (Supplementary Figure S7). The analysis with Jpred4 predicted that the insertion sequences form alpha helices, with a high probability to form a coiled-coil structure. Coiled-coil is a common structural motif in proteins, formed by two or more strands of alpha-helices winded around each other in superhelical fashion (Lupas, 1997; Truebestein and Leonard, 2016). The above mentioned amino acids enriched in the insertions are known to be involved in formation of the coiled-coil frequently, because of their propensity to form alpha-helices (Monera et al., 2002; Kwok and Hodges, 2004; Surkont and Pereira-Leal, 2015). All the insertion sequences were cut out and individually subjected to BLAST analysis, but no homologous sequence was identified outside the ArxA.

### Phylogenetic Reassessment of Partial *arxA* Gene Sequences Detected in Previous Studies

An additional phylogenetic analysis was performed to reassess the phylogenetic positions of previously reported partial sequences of the *arxA* gene. A neighbor-joining tree was reconstructed using a final dataset of 92 compared amino acid positions. The tree showed that all of the clones recovered from high-saline and high-pH samples are closely related to ArxA encoded in the genomes of haloalkaliphilic gammaproteobacteria (Supplementary Figure S8). The ArxA of *Desulfotomaculum* sp. TC-1 isolated from a hot spring was also related to these bacteria. In contrast, all the clones recovered from the Hot Creek riverbed sediment (HC) fell within a strongly supported cluster together with ARX of group 3 encoded in the genomes of bacteria isolated from the Jozankei hot spring (Supplementary Figure S8).

## Discussion

To the best of our knowledge, strain M52 is the first arsenite-oxidizing betaproteobacterium which possesses the *arx* genes. It was isolated from a microbial mat of a hot spring which contained approximately 3 mg/L of total arsenic (Kubota et al., 2010; Ospino et al., 2018). From the same mat, partial *arxA* gene sequences were detected with culture-independent methods (Ospino et al., 2018). The *arxA* gene of strain M52 was not detected in that analysis, suggesting that culture-based approaches are still effective to explore diversity of arsenite-oxidizing bacteria with *arx* genes. Although strain M52 lacks *aio* genes in the genome, it oxidized arsenite under microaerophilic and nitrate-reducing conditions. Some genomic features of the strain are consistent with these observations, as the genome harbors genes for respiration with oxygen and nitrate. The two types of terminal oxidases encoded in the genome are both high-affinity terminal oxidases characterized by low K_m_ values for oxygen (Morris and Schmidt, 2013), suggesting that the strain M52 is adapted to a low-oxygen environment. The concentration of oxygen in the air may be too high for this organism, and it might have suppressed arsenite oxidaion activity (Figure 2B). As another organism which oxidizes arsenite under microoxic conditions, *Hydrogenobaculum* strain H55 was reported in a previous study (Donahoe-Christiansen et al., 2004). In constrast to strain M52, strain H55 has genes for AIO (Clingenpeel et al., 2009).

The *arx* gene cluster of strain M52 had the same set of genes as those found in *A. ehrlichii* MLHE-1 and *Ectothiorhodospira* sp. PHS-1, with some small differences in positions of the genes. The comparative analysis showed that the majority of the genes in the *arx* gene cluster are conserved in more than half of the genomes (Figure 4). Especially, the *arxABC* genes encoding the putative functional elements of the enzyme are highly conserved. For other enzymes involved in arsenic metabolism, AIO arsenite oxidase and ARR arsenate reductase, the *aioBA* and *arrAB* genes encode the constituent subunits, and they are well-conserved in the genomes (Van Lis et al., 2013; Andres and Bertin, 2016). In contrast, the *arxC* gene homologous in the *arr* and *aio* gene cluster is not well-conserved (Slyemi and Bonnefoy, 2012; Grimaldi et al., 2013; Andres and Bertin, 2016). The *arxB2* gene, predicted to encode a type of ferredoxin protein, appears to be specific to the *arx* gene cluster, since no homologous genes have been described for AIO or ARR. A previous study demonstrated the expression of the *arxB2* gene in *Ectothiorhodospira* sp. BSL-9 (Hernandez-Maldonado et al., 2017). Additionally, transcripts were detected in the southern basin of Mono Lake (Edwardson and Hollibaugh, 2017). However, the role of *arxB2* in the arsenite oxidation remain unclear. The *arxD* gene was also highly conserved and predicted to encode a TorD-like protein required to introduce the cofactor into the enzyme (Genest et al., 2009). The occurrence of homologous genes in the *aio* or *arr* gene cluster (*aioD* and *arrD*) is variable as well (Van Lis et al., 2013; Andres and Bertin, 2016). The *arxXSR* genes are absent from the *arx* gene cluster of the genomes from members of the order *Oceanospirillales* (Figure 4). Similar findings were reported in previous studies on the gene clusters of *aio* and *arr*, which indicated that the presence of *aioXSR* and *arrXSR* is variable among taxonomic groups (Slyemi and Bonnefoy, 2012; Van Lis et al., 2013; Badilla et al., 2018). The *aioXSR* genes regulate the expression of the *aioAB* genes in some arsenite-oxidizing strains (Kashyap et al., 2006; Koechler et al., 2010; Sardiwal et al., 2010; Liu et al., 2012; Li et al., 2013). A bacterium which lacks the *aioXSR* module, *Halomonas* sp. HAL1, seems to employ another two-component system to regulate expression of *aioAB* (Chen et al., 2015). Thus far, the involvement of the *arxXSR* genes in regulation of the expression of *arxAB* has not been demonstrated.

The incongruency between the phylogeny of ARX and RPs suggested that horizontal gene transfer events have affected the evolution of the ARX and arsenite-oxidizing bacteria (Supplementary Figure S3). In other arsenic-related enzymes, the involvement of horizontal gene transfer in their evolutionary history has been reported as well (Andres and Bertin, 2016). In the genomes harboring *arx* genes, some features suggestive of gene transfer were identified. Some of the *arx* gene clusters were located in mobile genetic elements such as plasmids, as in *Sulfuricella denitrificans* skB26 (Watanabe et al., 2014) and *Halomonas* sp. A3H3 (NCBI Reference Sequence: NZ_HG423344.1) (Koechler et al., 2013), and in a genomic island in *Azoarcus* sp. CIB (Martin-Moldes et al., 2015). In strain M52, a gene encoding transposase (92% identity to the IS110 family transposase of *Thiomonas* sp.) was identified upstream of the *arxRSX*.

In this study, ARX were grouped into three groups based on phylogenetic analysis of ArxAB (Figure 6). The three groups have some specific characteristics, as follows. Strains with ARX in group 1 are all haloalkaliphiles and belong to the orders *Chromatiale* and *Oceanospirillales*. The phylogenetic analysis of previously reported data reconfirmed that the partial *arxA* sequences clustered with group 1 were all detected in environments with high pH and salinity. The sole exception was *Desulfotomaculum* sp. TC-1 obtained by PCR amplification (Wu et al., 2017). This strain exhibits optimum growth at pH 6.8 and belongs to the phylum *Firmicutes*, although *arx* genes identified in this study were only in the genomes of *Proteobacteria*. More detailed analysis of this organism is necessary to obtain the full sequences of the *arx* genes. *Ectothiorhodospira* sp. PHS-1 is haloalkaliphile belonging to the order *Chromatiale*, but its ARX was classified into group 2. Group 2 seemed to be the most heterogeneous group, because the genes encoding this type of ARX were identified in seven genomes belonging to four classes in the phylum *Proteobacteria*. These genomes included those of phototrophic haloalkaliphiles, heterotrophs growing in low-salt medium, and MAGs obtained from freshwater environments. Partial sequences of *arxA*, related to group 2, were detected in a freshwater lake and an alkaline salt lake. The former is Lake Mizugaki, from which some OTUs were obtained in this study, and the latter is Mono Lake, where close relatives of *Ectothiorhodospira* sp. PHS-1 were detected in previous studies (Figure 8). In contrast to these groups, ARX of group 3 was not identified in the genome of phototrophic organisms. The isolated strains with group 3 ARX are all sulfur-oxidizing chemolithoautotrophs. The MAGs with this type of ARX were obtained from a CO_2_-driven geyser (Probst et al., 2017) and groundwater sample (Anantharaman et al., 2016), suggesting that corresponding organisms do not depend on light. These findings also suggest that the ARX of group 3 is specific for low-salt environments. This idea is supported by phylogenetic analysis of the partial *arxA* gene sequences. Among the previously reported partial sequences, those from Hot Creek riverbed sediment were clustered with group 3 (Supplementary Figure S8). This sample is characterized by low salt concentrations in comparison to the other samples analyzed in the previous studies. The other partial sequences of group 3 were obtained in this study. Group 3 is characterized by a phylogenetic relationship that is highly congruent with the phylogeny of ArxD (Supplementary Figure S4).

The long insertion in ArxA was identified only in ARX of group 3 and encoded in the genomes of *Betaproteobacteria* and *Gammaproteobacteria*. The clone library analysis in this this study expanded knowledge about the diversity of ArxA with insertions (Supplementary Figure S8). It is important to point out that the primers used for this analysis were designed based on a limited number of group 3 ArxA sequences related to each other, and failed to amplify the *arxA* gene of strain M52 and J5B (Supplementary Figure S7). Therefore, the results have been affected by PCR bias and close relatives of strain M52 might be missed in the analysis. However, application of these primers resulted in the unexpected detection of *arxA* without insertions. One of the samples used in this analysis was the isolation source of strain M52, and the other one was used in a previous study to detect the *arrA* gene (Watanabe et al., 2017). In that study, arsenate respiration by *Sulfuritalea* in the deep water of Lake Mizugaki was suggested (Watanabe et al., 2017). In another sample of deep water obtained from the same lake in a different year, partial *arxA* gene sequences were detected with another primer pair (Ospino et al., 2018). It is plausible that anaerobic bacteria detected with forms of *arxA* and *arrA* are driving the arsenate cycle in the anoxic layer of water in this lake. In contrast to ARX of group 1 and group 2, involvement of the group 3 ARX in arsenite oxidation has not been demonstrated. Strain M52 is the first organism for which the presence of the genes for group 3 ARX and arsenite oxidation were both demonstrated (Figure 2, 3). The absence of the *aio* gene cluster in the genome of strain M52 suggests that the *arx* genes are responsible for its arsenite oxidation. However, further experimental evidence is required to confirm arsenite oxidation by group 3 ARX. Strain M52 also has a long insertion in ArxA and an unusual gene arrangement in the *arx* gene cluster characterized by the unique position of *arxB2* (Figure 4). The role of these elements in arsenite oxidation by strain M52 will be important subject of further studies.

## Author Contributions

MO, HK, and MF designed the study. MO and HK performed experiments and wrote the manuscript. MO conducted data analysis. All authors contributed to manuscript revision, and approved the submitted version.

## Conflict of Interest Statement

The authors declare that the research was conducted in the absence of any commercial or financial relationships that could be construed as a potential conflict of interest.

## Footnotes

1 ftp://ftp.ncbi.nlm.nih.gov/blast/db/

2 ftp://ftp.ncbi.nih.gov/genomes/genbank/bacteria/
