# Supplementary material for: Arsenite Oxidation by a Newly Isolated Betaproteobacterium Possessing arx Genes and Diversity of the arx Gene Cluster in Bacterial Genomes
Source: Front Microbiol. 2019 May 29;10:1210. doi: 10.3389/fmicb.2019.01210 (PMC6549141; doi:10.3389/fmicb.2019.01210)
Supplement: Supplementary file 1 [file Data_Sheet_1.PDF]

Supplementary Material

Arsenite oxidation by a newly isolated betaproteobacterium possessing *arx* genes and diversity of the *arx* gene cluster in bacterial genomes

Melody Cabrera Ospino*, Hisaya Kojima*, Manabu Fukui

The Institute of Low Temperature Science, Hokkaido University, Sapporo, Japan

*** Correspondence:**
Hisaya Kojima
kojimah@pop.lowtem.hokudai.ac.jp

Melody Cabrera Ospino
melodycabreraospino@gmail.com

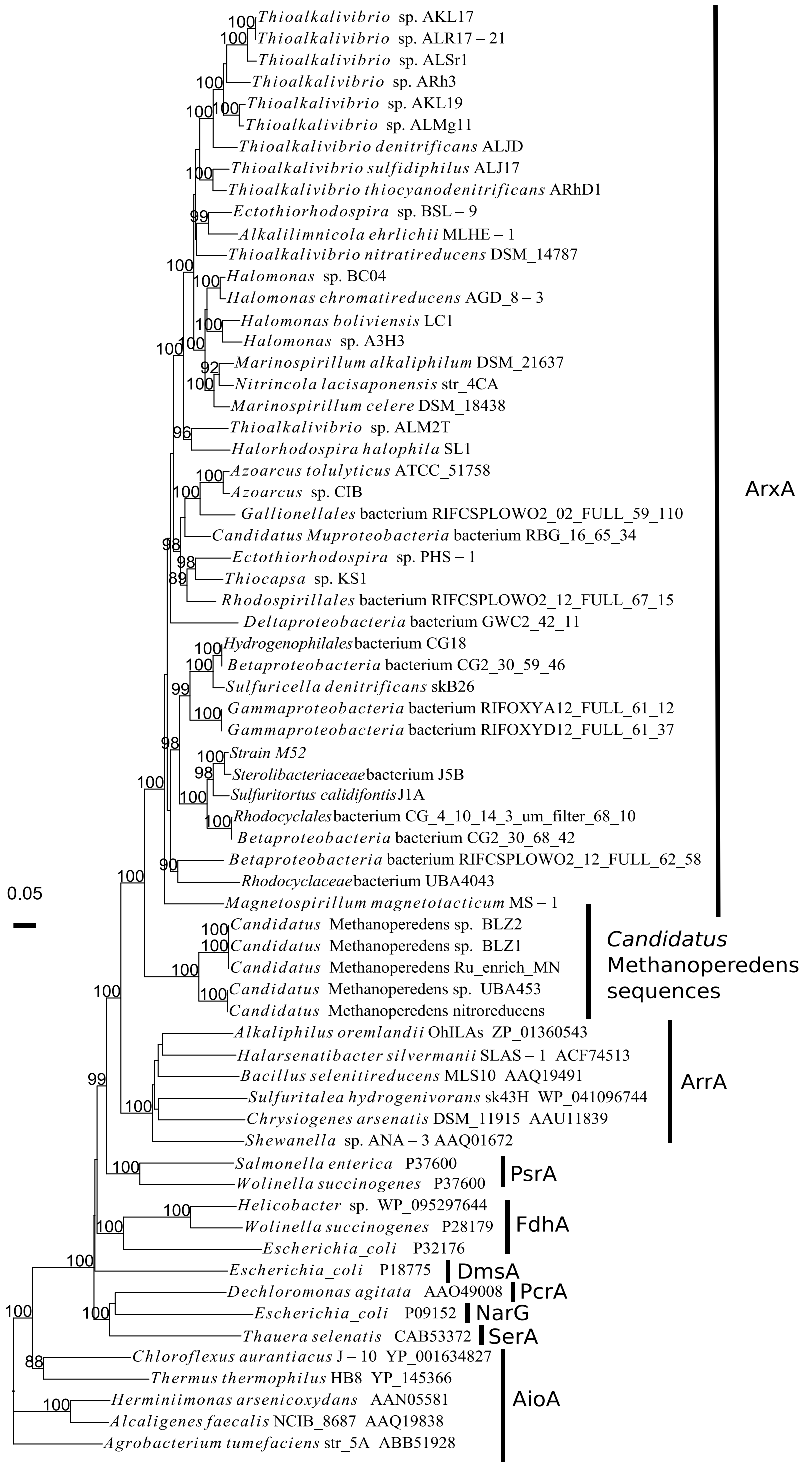

Supplementary Figure S1. Uncollapsed version of phylogenetic tree shown in Figure 5.

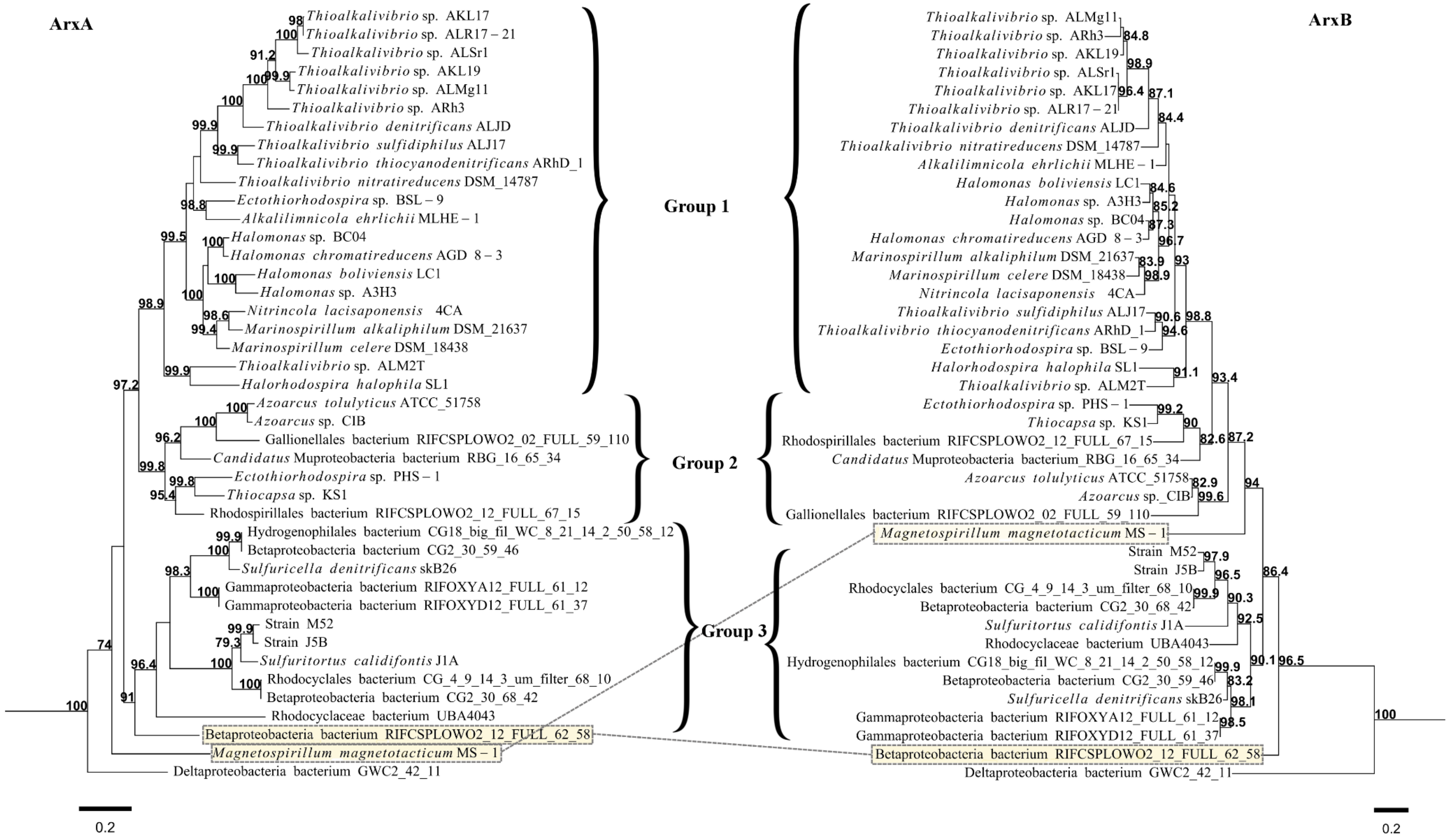

Supplementary Figure S2. Maximum-likelihood phylogenetic trees of ArxA and ArxB. Highlighted names connected by lines represent incongruent positions of same organisms in two trees. *Candidatus* Methanoperedens were used as outgroup. Support values above 70 are shown.

frontiers

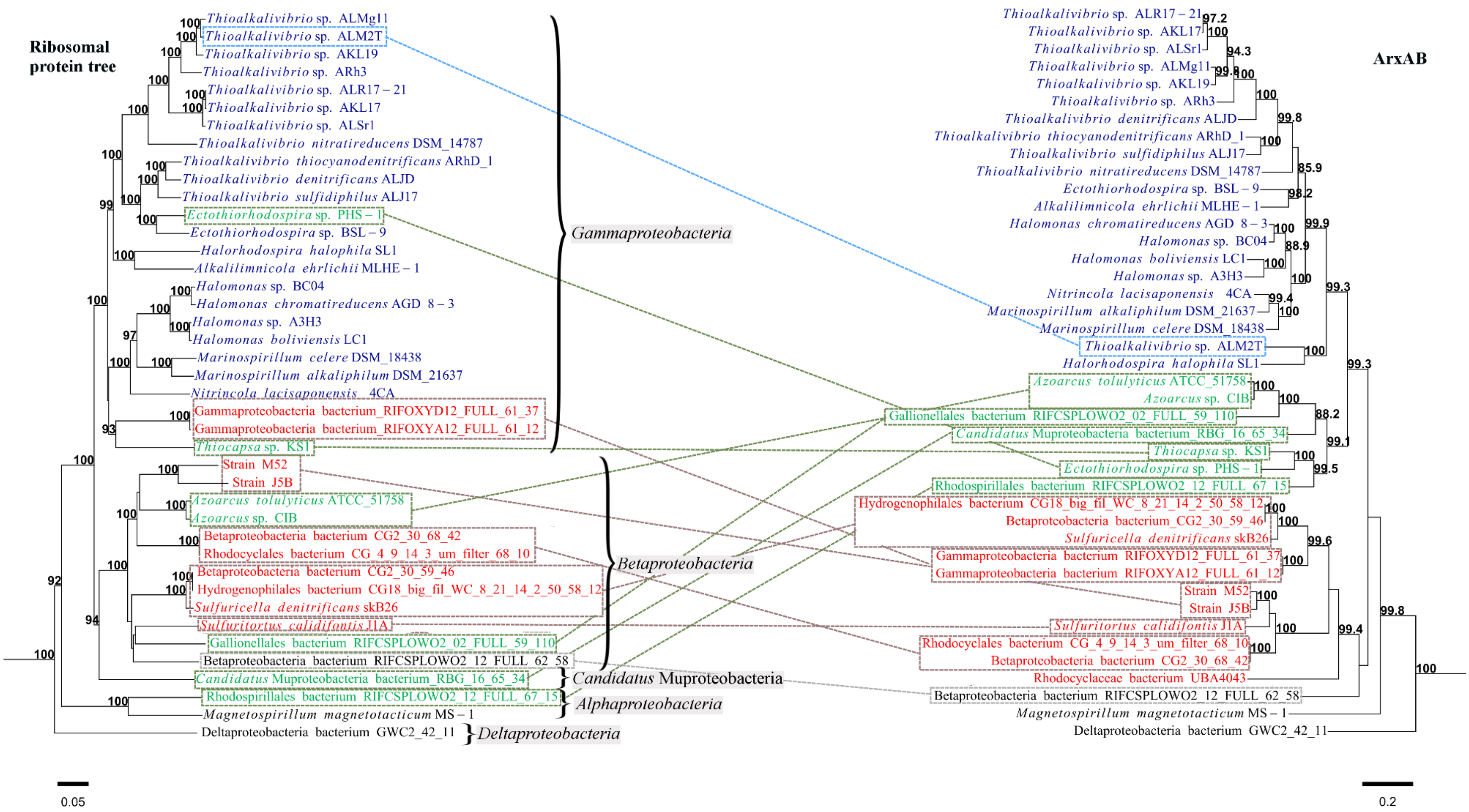

Supplementary Figure S3. Comparison between phylogenetic trees of ribosomal proteins (RPs) and ArxAB, shown in Figure 6 and Figure 7, respectively. Name of organisms with ARX of group 1, group 2, and group 3 are shown in blue, green and red respectively. Boxes connected by dashed lines highlight inconsistency between the trees.

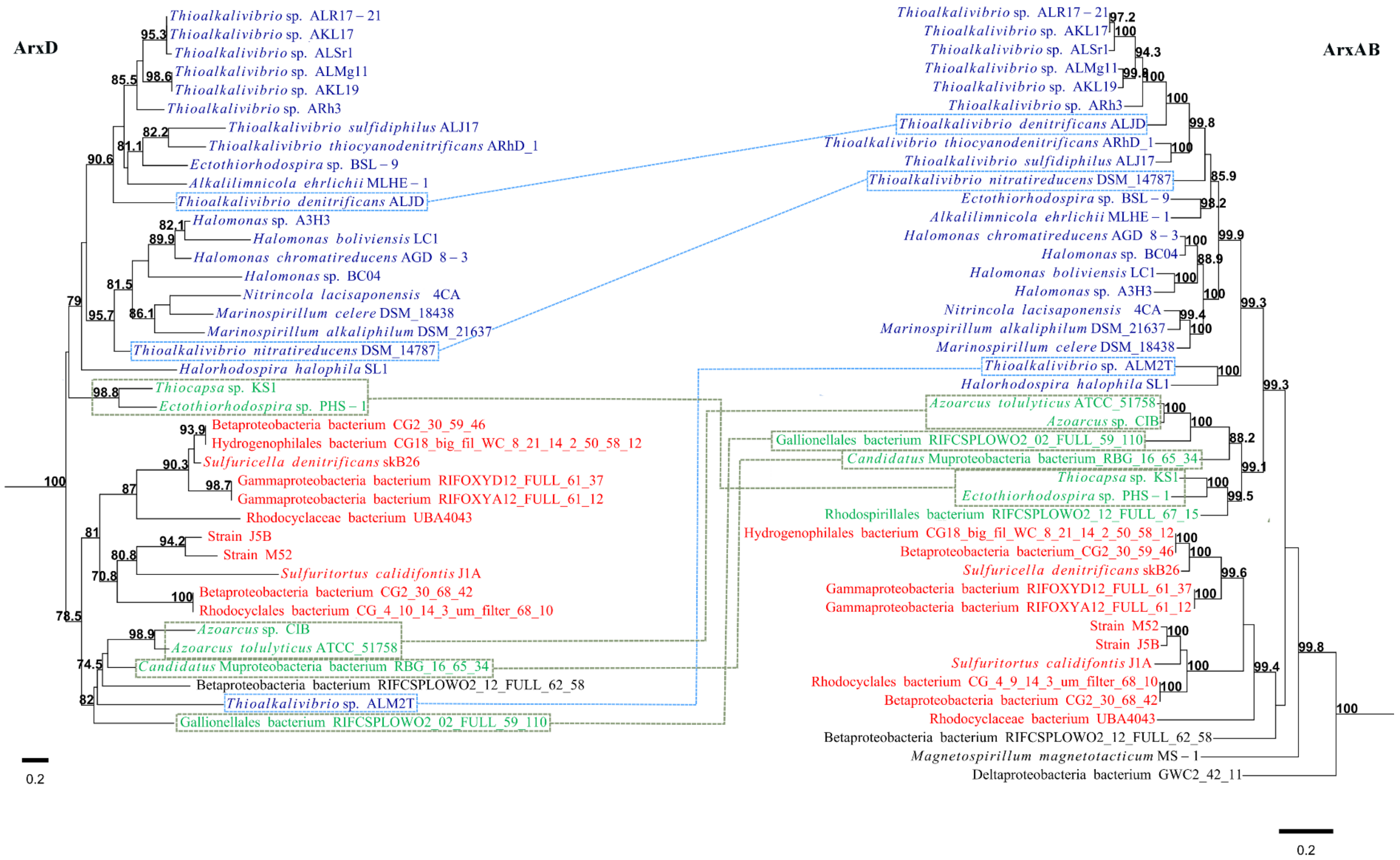

Supplementary Figure S4. Maximum-likelihood phylogenetic trees of ArxD, and comparison with the tree of ArxAB. *Candidatus* Methanoperedens were used as outgroup in both trees. Support values above 70 are shown. Name of organisms with ARX of group 1, group 2, and group 3 are shown in blue, green and red respectively. Boxes connected by dashed lines highlight inconsistency between the trees.

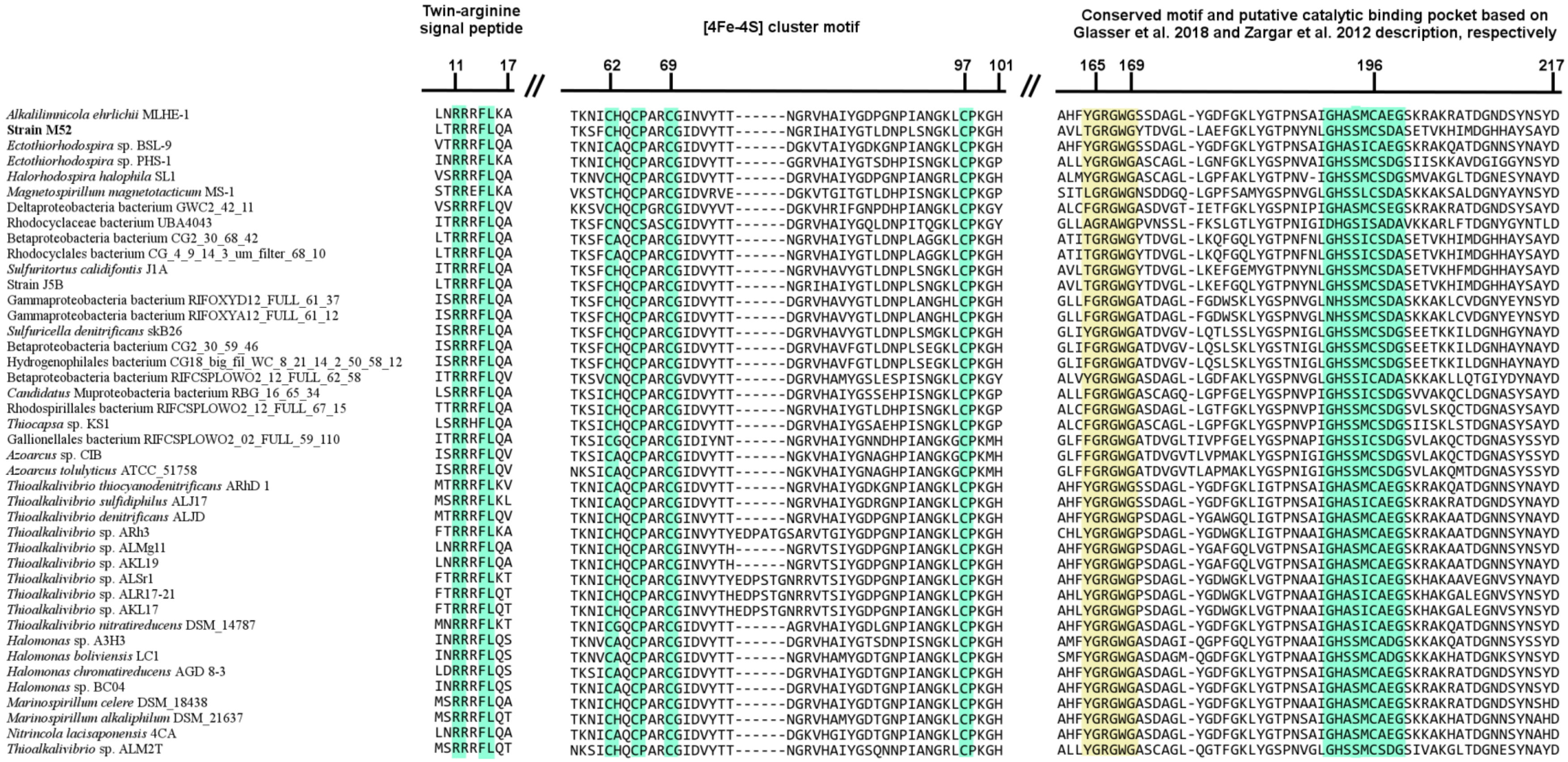

Supplementary Figure S5. Alignment of reference ArxA sequences collected in the present study, highlighting the conserved motif in the putative subunit A. The conserved motif were identified based on the described motif of the arsenite oxidase ArxA in *Alkalilimnicola ehrlichii* MLHE-1 and *Ectothiorhodosira* sp. PHS-1. Number in the upper part of the figure represents the position in the amino acid sequence of *Alkalilimnicola ehrlichii* MLHE-1.

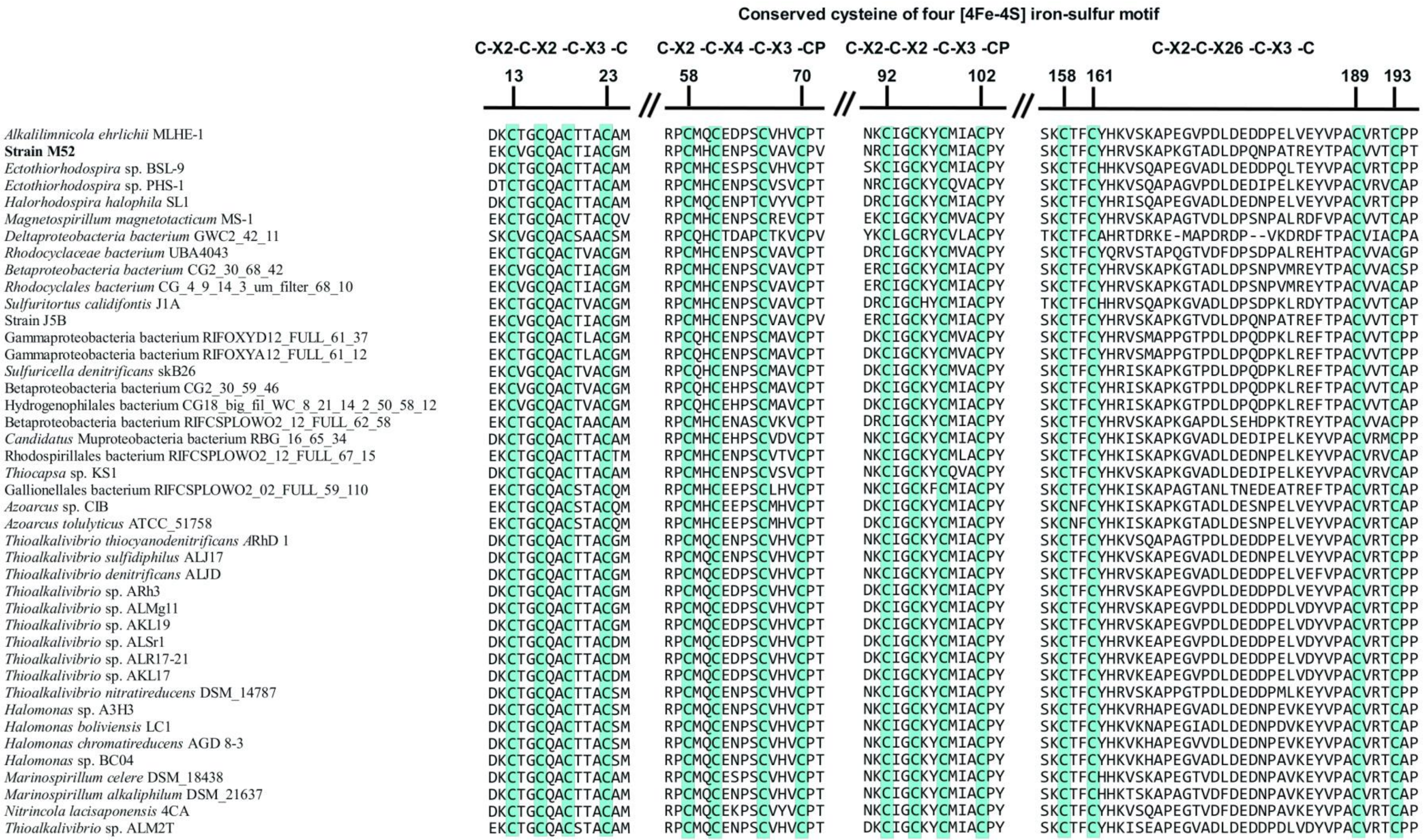

Supplementary Figure S6. Alignment of reference ArxB sequences collected in the present study, highlighting the conserved motif in the putative subunit B. The conserved motif were identified based on the described motif of the arsenite oxidase ArxB in *Alkalilimnicola ehrlichii* MLHE-1 and *Ectothiorhodosira* sp. PHS-1. Number in the upper part of the figure represents the position in the amino acid of *Alkalilimnicola ehrlichii* MLHE-1.

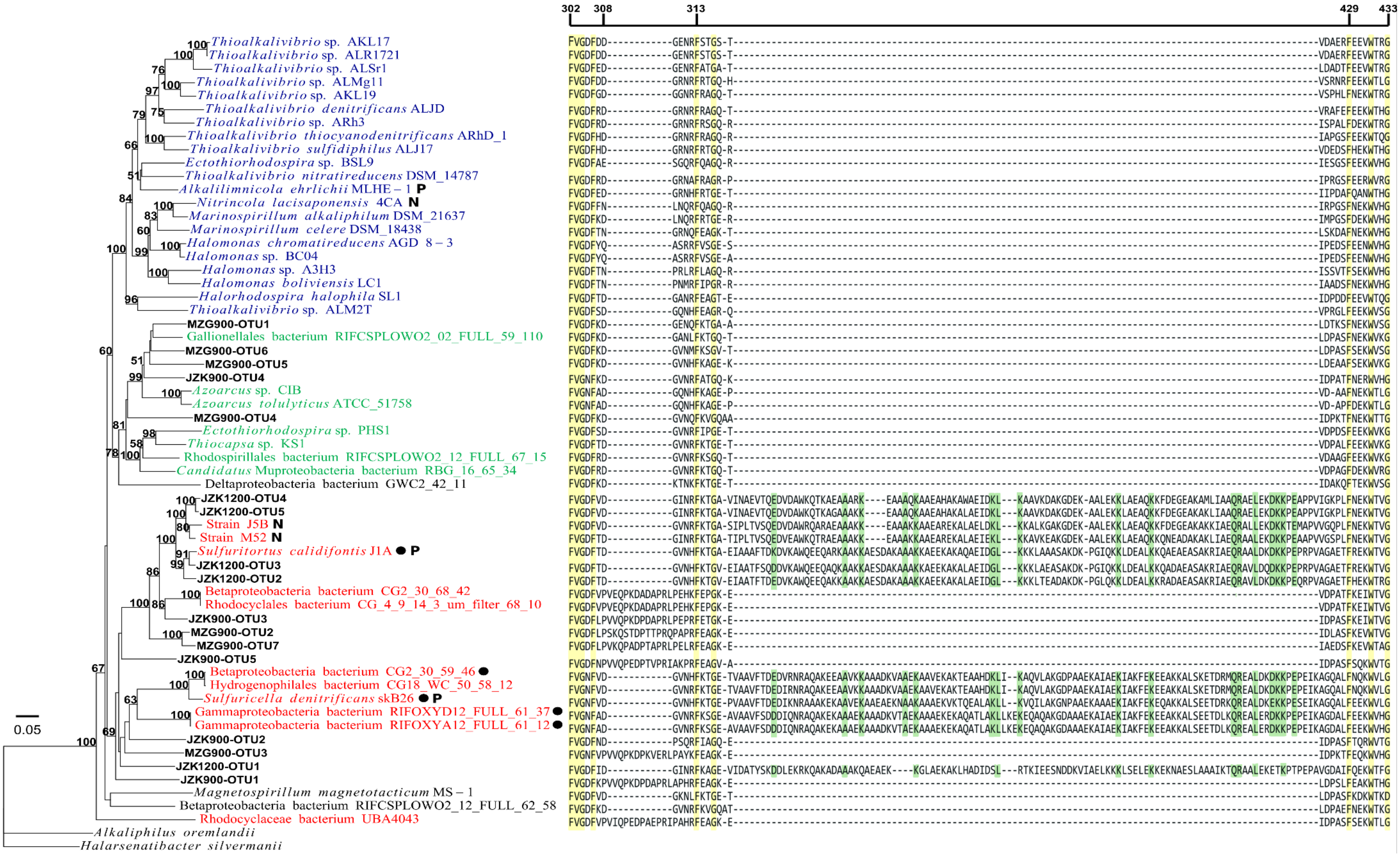

Supplementary Figure S7. Phylogenetic positions of ArxA sequences obtained in the clone libraries (JZK1200, JZK900 and MZG900) and distribution of insertion sequences within them. Phylogenetic tree was constructed using a dataset of 310 comparable amino acid positions. Arsenate respiratory reductase of *Alkaliphilus oremlandii* ABW18642 and *Halarsenatibacter silvermanii* ACF74513 were used as outgroup. Bootstrap values (1000 resampling) above 50 are shown. Black circles indicate the ArxA sequences used for the primer design. Genomic DNAs of organisms labeled with P and N were used to test PCR amplification with the new primer pair, resulting in positive and negative results respectively. In the sequence alignment, conserved residues within the insertion are highlighted with green, while those in all the sequences are shown in yellow. Numbers on the top indicate the position in the amino acid sequence of the ArxA from *Sulfuricella denitrificans* skB26. Dashes in the alignment indicate gaps.

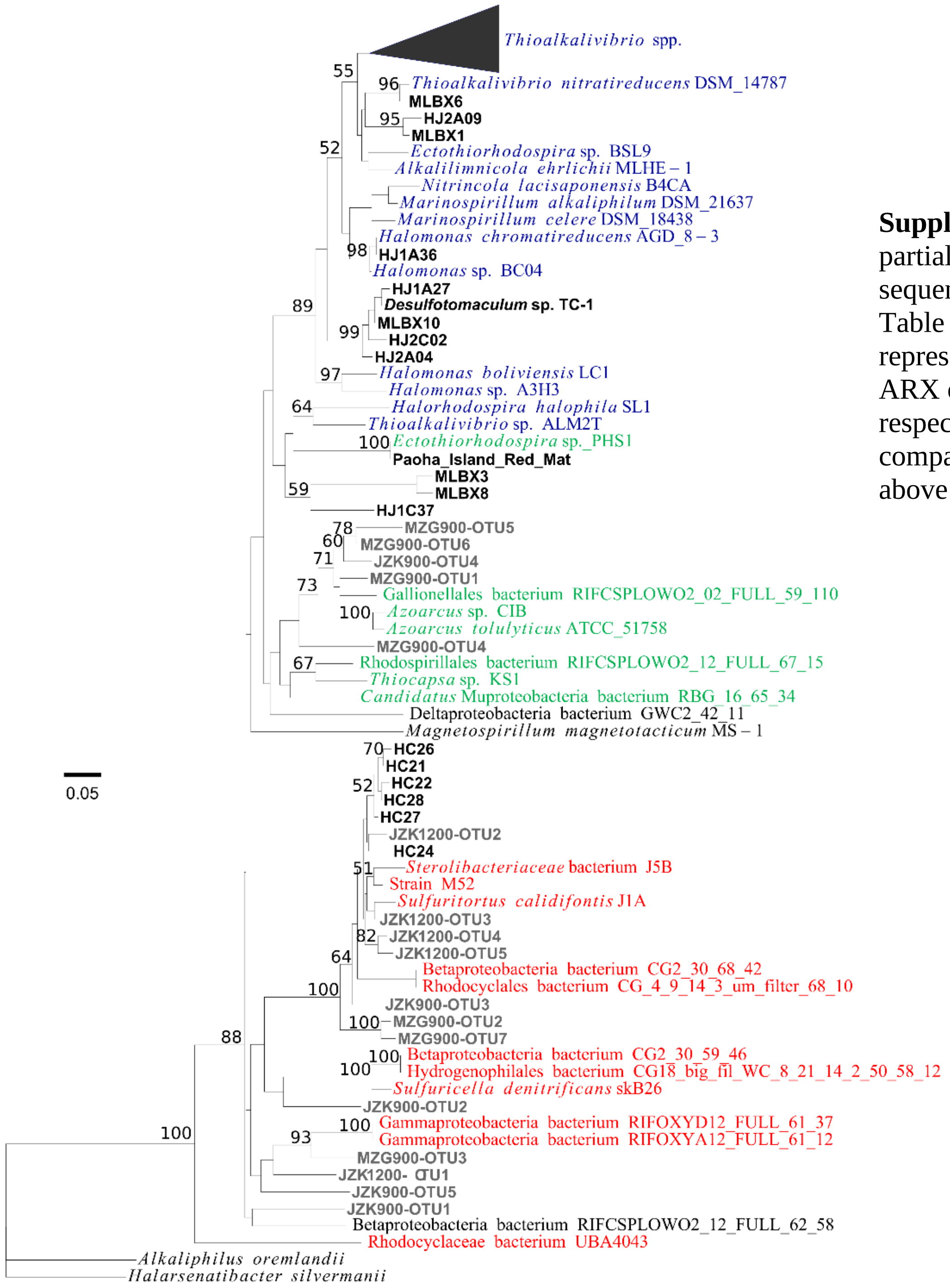

Supplementary Figure S8. Tree showing phylogenetic positions of partial *arxA* genes sequences. Names in bold black letters represent sequences reported in previous studies, and their details are shown in Table 1 except for *Desulfotomaculum* sp. TC-1. Names in gray letters represent the sequences obtained in this study. Name of organisms with ARX of group 1, group 2, and group 3 are shown in blue, green and red respectively. This tree was constructed using a dataset of 92 comparable amino acid positions. Bootstrap values (1000 resampling) above 50 are shown.
